# Development and validation of the Climate Model Confidence Index (CMCI): measuring ability to reproduce historical climate conditions

**DOI:** 10.1007/s00704-021-03581-5

**Published:** 2021-03-19

**Authors:** Micah J. Hewer, Nathan Beech, William A. Gough

**Affiliations:** Department of Physical and Environmental Sciences, University of Toronto, Scarborough, Canada

## Abstract

This study further develops and finally validates the Climate Model Confidence Index (CMCI) as a simple and effective metric for evaluating and ranking the ability of climate models to reproduce historical climate conditions. Modelled daily climate data outputs from two different statistical downscaling techniques (PCIC: Pacific Climate Impacts Consortium; SDSM: Statistical Down-Scaling Model) are compared with observational data recorded by Environment Canada weather stations located in Kelowna, BC (Canada), for the period from 1969 to 2005. Using daily data (*N* > 13,000), Student’s *t*-tests determined if there were statistically significant differences between the modelled and observed means while ANOVA *F*-tests identified differences between variances. Using aggregated annual data (*N* = 37), CMCI values were also calculated for the individual model runs from each statistical downscaling technique. Climate model outputs were ranked according to the absolute value of the *t* statistics. The 20 SDSM ensembles outperformed the 27 PCIC models for both minimum and maximum temperatures, while PCIC outperformed SDSM for total precipitation. Linear regression determined the correlation between the absolute value of the *t* statistics and the corresponding CMCI values (*R*^2^ > 0.99, *P* < 0.001). Rare discrepancies (< 10% of all model rankings) between the *t* statistic and CMCI rankings occurred at the third decimal place and resulted in a one rank difference between models. These discrepancies are attributed to the precision of the *t* tests which rely on daily data and consider observed as well as modelled variance, whereas the simplicity and utility of the CMCI are demonstrated by only requiring annual data and observed variance to calculate.

## Introduction

The earth’s climate is changing, and surface temperatures are rapidly increasing. According to the Intergovernmental Panel on Climate Change (IPCC 2019), since the pre-industrial period (1850–1900), land surface air temperatures have risen even faster than global surface air temperatures (which include air temperatures above both land and ocean surfaces). Comparing 1850–1900 with 2006–2015, mean land surface air temperatures have increased by 1.53 °C while mean global surface air temperatures have increased by 0.87 °C (IPCC 2019). This observed warming has resulted in an increased frequency, intensity and duration of heat-related events, including heatwaves over most land regions (IPCC 2019). While the frequency and intensity of droughts have increased in some regions, there has also been an increase in the intensity of heavy precipitation events on a global scale (IPCC 2019). Global warming has led to shifts of climate zones in many world regions, including the expansion of arid climate zones and the contraction of polar climate zones, which has led to changes in the ranges and abundances of plant and animal species as well as shifts in their seasonal activities (IPCC 2019).

Canada’s climate is no exception to this global phenomenon, but rather, has been warming at an even greater rate in many regions across the country. A reconstruction of global surface air temperatures from 1901 to 2012 by Vose et al. (2012) found that the greatest warming across the globe has occurred over northwestern North America and over central Eurasia. In Canada, significant warming trends in annual mean temperatures ranging from 1.8 to 3.8 °C were reported by Vincent et al. (2015), within almost every region across the nation from 1948 to 2012. The observed anomalies averaged over the country indicate a significant increase of 1.78 °C over the past 65 years (Vincent et al. 2015). Precipitation totals have also increased across Canada, principally in the northern areas and across all four seasons (Vincent et al. 2015). However, winter season precipitation has decreased in the southwestern region of Canada (British Columbia and Alberta), and there have been widespread decreases in the amount of precipitation falling as snow across the southern regions of Canada (Vincent et al. 2015). Furthermore, spring precipitation has been shifting from snow to rain across Canada and the duration of snow cover has also been decreasing (Brown et al. 2010; Mekis and Vincent 2011).

Due to regional and activity-specific differences, Warren and Lemmen (2014) conclude that climate change can have both positive and negative effects on Canadian communities and economies. Vincent et al. (2018) suggest that longer and warmer growing seasons across Canada may allow crops to be grown further north (where soil conditions permit). Campbell et al. (2014) indicate that outdoor feeding seasons for livestock may also expand in a warmer climate. However, a warmer climate in Canada may introduce new pests and diseases as well that could negatively affect agriculture (Campbell et al. 2014). Blankinship and Hart (2012) suggest that declining snowfall and reduced snowpack in a warmer climate may reduce water availability for agriculture, especially in southern regions of Canada. While Casati et al. (2013) advise that a warmer climate is characterised by more hot days and hot nights which are associated with negative human health effects including heat-related human mortality, Berardi and Jafarpur (2020) project decreased energy demand for heating buildings during warmer winters in Canada but project an equal or greater increase in energy demand for cooling buildings during hotter summers. Increased freeze-thaw days (that is, more days with daytime temperatures above 0 °C, while nighttime temperatures remain below freezing) in central Canada (Vincent et al. 2018) may have a negative impact on road maintenance (Hershfield 1979; Schmidlin et al. 1987; Ho and Gough 2006). However, positive impacts on road maintenance due to fewer freeze-thaw days may be experienced in other areas of the country, such as BC, Ontario, Quebec and the Maritime provinces (Vincent et al. 2018). A warmer climate in Canada could be beneficial for shipping due to less ice coverage on lakes and other water passages (Hewer and Gough 2019a); however, warming temperatures can also limit transportation in areas that depend on ice roads (Hori et al. 2017, 2018). Furthermore, a warmer climate can extend camping seasons (Hewer et al. 2016; Hewer and Gough 2019b) and other warm weather tourism activities (Hewer and Gough 2016a, b), but will be detrimental to many winter season tourism activities, especially those dependent on snow and ice (Hewer and Gough 2018).

The activity being assessed and the location of the assessment often determine whether climate change impacts will be beneficial or detrimental. Some activities will see greater opportunities while the sustainability of others will be threatened, and those same activities may be affected differently across various locations. For this reason, activity specific, locally focused, climate change impact assessments (CCIAs) are of great importance and utility for adaptation planning and policy development. Global climate models (GCMs) provide important information regarding how large-scale climate conditions are likely to change over the course of the 21^st^ century (Taylor et al. 2012); however, activity specific, locally focused CCIAs require climate change projections at finer temporal (Zhang et al. 2011) and spatial (Murdock et al. 2016; Kopparla et al. 2013; Salathe et al. 2007) scales. For this reason, downscaling of GCM projections is a vital step within CCIA (Hewer et al. 2016; Hewer and Gough 2016a, 2020; Hewer and Brunette 2020). Downscaling uses large-scale atmospheric variables to predict local meteorological conditions, which is appealing to CCIA due to the added details that inform site-specific assessment and management of climatic risk (Bürger et al. 2012; Sobie and Murdock 2017). This can be done using dynamical downscaling resulting in regional climate models (Mearns et al. 2012) or through statistical downscaling to create local point, daily timescale climate change scenarios (Wilby et al. 2004). Depending on the scale and scope of the exposure unit being assessed, and the resources available to the researchers, either downscaling approach may be appropriate for a given CCIA. Regardless, decisions often must be made around which GCM outputs to use, or which RCMs to rely upon, or which statistically downscaled climate scenario to base projected impacts upon. It is this need that led to the creation of the Climate Model Confidence Index (CMCI), initially conceived to guide decisions around which GCMs to use in CCIA (Hewer and Gough 2016a, b, 2019a, b, 2020; Hewer and Brunette 2020); but through the current study, now shows utility for comparing statistical downscaling techniques, and for selecting specific model runs from among statistically downscaled climate change scenarios.

To further illustrate the utility of a metric like the CMCI, there are 41 GCMs available as part of the Coupled Model Intercomparison Project Phase 5 (CMIP5), but which model output should researchers use to inform a CCIA? Some have suggested using a “full ensemble” of all GCM outputs by averaging the anomalies across each model (Fenech et al. 2007; IPCC 2010; Hewer et al. 2016). While others have been more inclined to try and capture the full range of uncertainty associated with future climates by selecting the two models that represent the least and greatest projected change (Scott et al. 2002, 2003). Conversely, Hewer and Gough (2016a, b, 2019a, b, 2020; Hewer and Brunette 2020) used the CMCI to rank the available GCMs from CMIP5, based on their ability to reproduce historical climate conditions, then selected the top three models and created a “selective ensemble” by averaging the anomalies across those three models. Nonetheless, the “full” versus “selective” ensemble debate becomes a moot point when deciding which statistically downscaled scenario to use in a CCIA because you cannot average the daily outputs from across different models or model runs without losing the natural day-to-day variability associated with the local climatology (Gough 2008). Thus, when deciding which statistical downscaling technique, or which specific model run to use, the CMCI is a useful tool within the practice of CCIA.

This paper will present the CMCI as a tool for measuring the ability of climate models to reproduce historical climate conditions. It will review the way in which the CMCI has been previously measured then present a newly devised scheme for the interpretation of CMCI values. The CMCI, which only requires average annual climate data for its calculation, will then be validated using daily observational data and established statistical tests, based on a case study approach for comparing the performance of two statistical downscaling techniques in Kelowna, BC, Canada, a town centred within the Okanagan Valley. Finally, the utility of this metric and its limitations will be discussed.

## Methods

### Goal and objectives

The purpose of this study is to further develop and finally validate the Climate Model Confidence Index (CMCI). This study goal will be guided by the following research objectives:

- Determine historical climate conditions (minimum and maximum temperatures as well as total precipitation) for Kelowna, BC; based on daily weather station data recorded by Environment Canada (available from January 1969 to December 2019).
- Access statistically downscaled historical climate conditions for Kelowna, BC, from the Pacific Climate Impacts Consortium (PCIC) at the University of Victoria (PCIC’s historical baseline runs from 1950 to 2005).
- Perform statistical downscaling using the Statistical Down-Scaling Model (SDSM) to reproduce historical climate conditions in Kelowna, BC for the overlapping historical time-period covered by both the Kelowna weather stations and the PCIC historical baseline (1969 to 2005).
- Evaluate and compare the ability of both PCIC and SDSM to reproduce historical daily climate conditions in Kelowna, BC, by using Student’s *t* tests to determine if there are significant differences between modelled and observed means as well as ANOVA *F*-tests to determine if there are significant differences between modelled and observed variances.
- Finally, calculate the CMCI and compare its ranking of PCIC models and SDSM ensembles to the results from the *t* tests to determine if the index ranks climate models in a similar way, while also employing linear regression analysis to determine correlation between the two measures.

### Study area

The Okanagan Valley in British Columbia is an area of considerable social, economic and environmental significance within a Canadian context. The valley is a major agricultural area within the province, associated with the growth and production of numerous fruit crops including apples (Caprio and Quamme 1999), grapes (Caprio and Quamme 2002), pears (Quamme et al. 2010), as well as apricots, peaches and cherries (Caprio and Quamme 2006). This location is also unique climatologically. The Okanagan Valley is located within the south-central region of the Canadian province British Columbia. This long narrow valley is approximately 300 km east of the Pacific Ocean, beginning at the USA/Canada border and running northward for approximately 160 km. The region lies in a rain shadow between two north-south trending mountain ranges, resulting in low annual average precipitation that is distributed evenly throughout the year, with only modest winds being typical (Rayne and Forest 2016). Summers in the Okanagan Valley are hot, with long day-light hours and high light intensity, being associated with average temperatures around 21 °C and maximum temperatures reaching 40 °C, including prolonged periods with days above 30 °C (Rayne and Forest 2016). Winters are generally cold, with temperatures dropping below freezing for long periods, and with extreme cold events bringing temperatures as low as − 25°C (Rayne and Forest 2016). However, the valley’s extensive lakes are key physiographic features responsible for moderating the otherwise mountainous/continental climate that is typically characterised by extreme heat in the summer and extreme cold in the winter (Rayne and Forest 2016). Kelowna is a town located in the centre of the Okanagan Valley. This site was selected to represent the region due to its central location and the availability of a long historical climate record from the Environment Canada weather stations located there (1969 to the present day).

### Statistical downscaling

The Pacific Climate Impacts Consortium (PCIC) at the University of Victoria offers statistically downscaled daily Canada-wide climate scenarios, at a gridded resolution of approximately 10 km for the simulated period of 1950–2100, where 1950 to 2005 represents the historical baseline. These statistically downscaled outputs are based on Global Climate Model (GCM) projections from the Coupled Model Intercomparison Project Phase 5 (CMIP5) (Taylor et al. 2012), along with historical daily gridded climate data for Canada (McKenney et al. 2011; Hopkinson et al. 2011). PCIC acknowledges that gridded values may differ from local climate stations and biases may be present at high elevations or in areas with low station density (Eum et al. 2014). PCIC downscales GCM data to a finer resolution using two different methods: the first is Bias-Correction Spatial Disaggregation (BCSD) (Wood et al. 2004), following the modifications proposed by Maurer and Hidalgo (2008), which include the incorporation of minimum and maximum temperature instead of mean temperature, as suggested by Bürger et al. (2012) and bias correction using detrended quantile mapping with delta method extrapolation (Bürger et al. 2013). The BCSD downscaling algorithm has been analysed and validated for British Columbia by Werner (2011), Bürger et al. (2012, 2013) as well as Werner and Cannon (2016). In addition to BCSD, PCIC projections are also available using Bias Correction/Constructed Analogues with Quantile mapping reordering (BCCAQ). BCCAQ is a hybrid method that combines results from BCCA (Maurer et al. 2010) and quantile mapping (QMAP) (Gudmundsson et al. 2012). According to PCIC (2021), BCCA uses similar spatial aggregation and quantile mapping steps as BCSD but obtains spatial information from a linear combination of historical analogues for daily large-scale fields, avoiding the need for monthly aggregates. Furthermore, PCIC (2021) states that QMAP applies quantile mapping to daily climate model outputs that have been interpolated to the high-resolution grid using the climate imprint method of Hunter and Meentemeyer (2005). Historical climate data (1950 to 2005) for each of the available 27 GCMs was retrieved from the PCIC website for the grid box associated with Kelowna, BC, Canada (119.38° W, 49.96° N) (https://data.pacificclimate.org/portal/downscaled_gcms/map/).

The Statistical DownScaling Model (SDSM) introduced by Wilby et al. (2002) is a hybrid of the stochastic weather generator and transfer function methods. SDSM uses large-scale circulation patterns and atmospheric moisture variables to condition local-scale weather generator parameters (Wilby et al. 2002). Additionally, stochastic techniques are used to artificially inflate the variance of the downscaled daily time series to better accord with site-specific observations (Wilby et al. 2002). The theoretical origins of SDSM lie in a series of papers that explored statistical relationships between indices of atmospheric circulation and local meteorology (Wilby 1994, 1995, 1997, 1998; Conway et al. 1996). The SDSM algorithm (Narula and Wellington 1977) is a conditional weather generator since atmospheric circulation indices and regional moisture variables are used to estimate time-varying parameters describing daily weather at individual sites (Wilby and Dawson 2013). SDSM relies on reanalysis data from the National Centers for Environmental Prediction and the National Center for Atmospheric Research, which represents the state of the Earth’s atmosphere during the baseline time-period, to identify relevant atmospheric predictor variables and create predictive models to generate synthetic weather data capable of reproducing past climate conditions for a specific location (Wilby et al. 2002). From 2001 to 2012, over 170 studies were documented using SDSM within the field of applied climatology (Wilby and Dawson 2013), not including more recent publications such as several CCIAs (Hewer et al. 2016; Hewer and Gough 2016a, 2020; Hewer and Brunette 2020). NCEP data was downloaded from the SDSM website for the grid box associated with Kelowna, BC (https://sdsm.org.uk/data.html). SDSM was then calibrated with this NCEP reanalysis data and the weather station data from Environment Canada’s Kelowna weather stations. Kelowna station data was retrieved from Environment Canada’s historical climate archive for the period from when the record began in 1969 up to the present day (https://climate.weather.gc.ca/historical_data/). The historical baseline within SDSM was then set to match the overlapping time period between both the station data (1960 to 2019) and the PCIC historical baseline (1950 to 2005), resulting in a common baseline period from 1969 to 2005 (37 years).

### Evaluating ability to reproduce historical climate

Both PCIC and SDSM generated synthetic climate data at the daily timescale for the period from 1969 to 2005 for maximum temperatures (*T*_max_), minimum temperatures (*T*_min_) and total precipitation (*P*_tot_). This modelled data was then compared with the observational data recorded at the Kelowna weather stations. The 37 years of observational data, along with the modelled SDSM data (including 20 individual ensembles), contained a total of 13,514 days. However, due to differences in the ways various GCMs define year lengths: 360 day years, 365 day years and calendar years (which include leap days), the modelled outputs from PCIC for this same 37-year time-period varied in length from 13,320 days (360 day year), to 13,505 days (365 day year), to 13,514 days (calendar year). The modelled data for the 27 different PCIC models and the 20 different SDSM ensembles were then compared with the observational record to evaluate the performance of these two statistical downscaling techniques regarding their ability to reproduce historical climate conditions in Kelowna, BC. It is generally accepted that climate models can be evaluated based on their ability to reproduce historical climate conditions (Randall et al. 2007) and acknowledged that some models perform better in certain regions than they do in others (Macadam et al. 2010). Student’s *t* tests were used to determine if there were statistically significant differences at the 95% confidence level (*P* < 0.05) between the modelled and observed means, based on the daily data from 1969 to 2005 (*N* = 13,320 to *N* = 13,505). The formula for the two sample *t* tests assuming unequal variances used in this study was as follows:

$$ t=\frac{\mu_{\mathrm{obs}}-{\mu}_{\mathrm{mod}}}{\sqrt{\frac{\sigma_{\mathrm{obs}}^2}{N_{\mathrm{obs}}-1}+\frac{\sigma_{\mathrm{mod}}^2}{N_{\mathrm{mod}}-1}}} $$

The *t* statistic can be either negative or positive, indicating whether the modelled data overestimated (−) or underestimated (+) baseline conditions, when compared with the observational data. The absolute value of the *t* statistic (│*t*│) was then used to ignore the direction of the recorded difference and rank the ability of these PCIC models and SDSM ensembles for reproducing local climate conditions, where the lower that │*t*│ was, the closer the modelled data was to the observational record. Furthermore, ANOVA *F*-tests were used to determine if there were statistically significant differences between the modelled and observed variances within the daily data for this same baseline time-period. The formula for the two sample *F*-tests used in this study were as follows:

$$ F=\frac{\sigma_{\mathrm{mod}}^2}{\sigma_{\mathrm{obs}}^2} $$

The *F* statistic is centred on the value of 1, where if the modelled variance is equal to the observed variance, then the *F* statistic is equal to 1. Whereas if the modelled variance is greater than the observed variance, then the *F* statistic will be less than 1, and if the modelled variance is less than the observed variance, then the *F* statistic will be greater than 1. Finally, the magnitude of departure away from 1 (whether above or below) indicates the significance of the differences between the observed and modelled variance and is expressed by the *P* value, where when *P* < 0.05 differences were considered statistically significant at the 95% confidence level.

### Climate Model Confidence Index

#### Measurement

The Climate Model Confidence Index (CMCI) has its origins traced back to a doctoral thesis at the University of Toronto (Fenech 2009). The index was subsequently taught for several years to follow as a metric for evaluating and ranking GCM performance for use in CCIAs during graduate courses within the Department of Physical and Environmental Sciences at the University of Toronto (Canada). However, the index was not formally published within the academic literature until it was utilised as part of a series of CCIAs conducted by Hewer and Gough (2016a, b), where it first appeared under the name of the Gough-Fenech Confidence Index (GFCI). Hewer and Gough (2019a, b, 2020) continued to champion the use of this index in what was referred to as the “selective ensemble” approach for creating GCM climate change projections and subsequently renamed it the Global Climate Model Confidence Index (GCM-CI). The rationale behind the creation and use of the GCM-CI was again that climate models can be evaluated based on their ability to reproduce baseline climate conditions and that some models perform better in certain regions than others (Randall et al. 2007; Macadam et al. 2010). The GCM-CI was therefore used to evaluate and rank GCMs available within CMIP5 and create selective ensembles of GCM outputs to generate climate change projections for a particular region, or for a specific location (in which case, GCM outputs were then used to force statistically downscaled climate change scenarios). Since this paper is not evaluating the ability of GCMs to reproduce historical climate conditions, but rather, the ability of statistical downscaling techniques to do the same (although still validating the metric for use on both scales), the index name has been shortened to the Climate Model Confidence Index (CMCI). We believe this name is more encompassing of the index’s utility and also hope will stand as its final name of reference. Regardless of the name of reference or the publication in which it appeared, the formula of the CMCI has always remained the same:

$$ \mathrm{CMCI}=\frac{\left|{\mu}_{\mathrm{obs}}-{\mu}_{\mathrm{mod}}\right|}{\sigma_{\mathrm{obs}}} $$

Calculation of the CMCI simply requires taking the absolute value of the observed baseline mean minus the modelled baseline mean then dividing by the standard deviation of the annual means over that same baseline time-period; which in this case was 1969 to 2005 (*N* = 37), but elsewhere has always been 1981 to 2010 (*N* = 30). The CMCI numerator is derived from the mean absolute error (MAE: Willmott and Matsuura 2005), an established method for evaluating the ability of modelled predictions to replicate observational data, while the CMCI denominator is derived from the process of standardization (*Z* score: Clark-Carte 2014), which allows for comparison of CMCI values across difference units of measurement (e.g. temperature and precipitation). Through the process of this study, we concluded that it is critical for the CMCI to be calculated with the aggregate terms described above and does not perform the same way if it is calculated at the daily time scale, primarily because the standard deviation of daily data is not as meaningful as the standard deviation of annual data (from a climate perspective), but also because daily total precipitation data does not follow a normal distribution while annual total precipitation data does.

#### Interpretation

From 2016 to 2020, while the CMCI was under development and being utilized within published CCIAs, there was no clear means devised by which to interpret the resulting values, other than to state that the lower the value the better the model performance is in relation to reproducing historical climate conditions. The current study sets forward the following four categories associated with CMCI values to describe the ability of climate models to reproduce historical climate conditions in a particular location: “good” (CMCI: 0.00 to 0.25), “satisfactory” (CMCI: 0.26 to 0.50), “poor” (CMCI: 0.51 to 1.00) and “unacceptable” (CMCI > 1.00). Previously, the index has always been used to evaluate and rank GCMs and then select and average the outputs from the best three (those with the lowest three values) to create a “selective ensemble” (Hewer and Gough 2016a, b, 2019a, b, 2020). However, over the years, thought had been given to expanding the selection process to include more GCMs within the ensemble through the process of exclusion rather than selection (i.e. rather than selecting the best three, exclude the worst or unacceptable models), thereby creating what would have been referred to as an “optimal ensemble” (Hewer and Brunette 2020). The main barrier to this development was the lack of a clear indication of which models were acceptable and which models were unacceptable, for a particular study area; and because no consensus was formed, the projects often defaulted back to the selective ensemble approach. Furthermore, because these previous studies were always working with averages over the 30-year baseline or annual averages and the standard deviation of those annual averages, there was little ability to further develop the index or validate its results against more robust statistical methods. However, the current study was able to compare the results and ranks associated with │*t*│ for each of the 27 PCIC models and each of the 20 SDSM ensembles with the corresponding CMCI values. It is from this process that the “good” category was defined in that this was the range of CMCI vales, on average, associated with *t* statistics that demonstrated no statistically significant differences (at the 95% confidence level), between the modelled and observed baseline.

The rationale behind the other three categories is based on the calculation of the CMCI itself and the premise that it is a ratio value describing what percentage of the standard deviation of annual averages over the baseline period is represented by the absolute value of the differences between modelled and observed baseline means. Therefore, CMCI values ≤ 0.50 are considered “satisfactory” in that the difference between modelled and observed baseline means is less than or equal to half the standard deviation of annual means (thereby representing natural interannual climate variability across the baseline period). CMCI values > 0.50 but ≤ 1.00 are considered “poor” (yet still acceptable, by implication), in that the differences between means (whether positive or negative) are greater than half but less than one standard deviation away from the interannual mean. Finally, CMCI values > 1.00 were considered “unacceptable” in that the differences between modelled and observed baseline means were greater than the standard deviation of annual means during that period (therefore being considered outside the scope of historical interannual climate variability).

#### Validation

The ranking of PCIC models and SDSM ensembles based on CMCI values was finally compared with the rankings based on │*t*│, thus validating the index as a simple and accurate method for evaluating the ability of climate models to reproduce historical climate conditions. The accompanying ANOVA *F*-tests demonstrating if there were statistically significant differences between observed and modelled variances were not used to rank the models, but rather, in an effort to further examine any potential discrepancies between the ranking of models based on │*t*│ and the ranking of models based on CMCI values. Finally, the coefficient of determination (*R*^2^) from linear regression analysis was also used to determine the relationship between │*t*│ and corresponding CMCI values between the PCIC models and the SDSM ensembles for each climate variable. High correlations (*R*^2^ > 0.95) between these two measures of the ability of climate models to reproduce baseline conditions would thereby further validate this metric.

## Results

### Maximum temperature

**Fig. 1:**
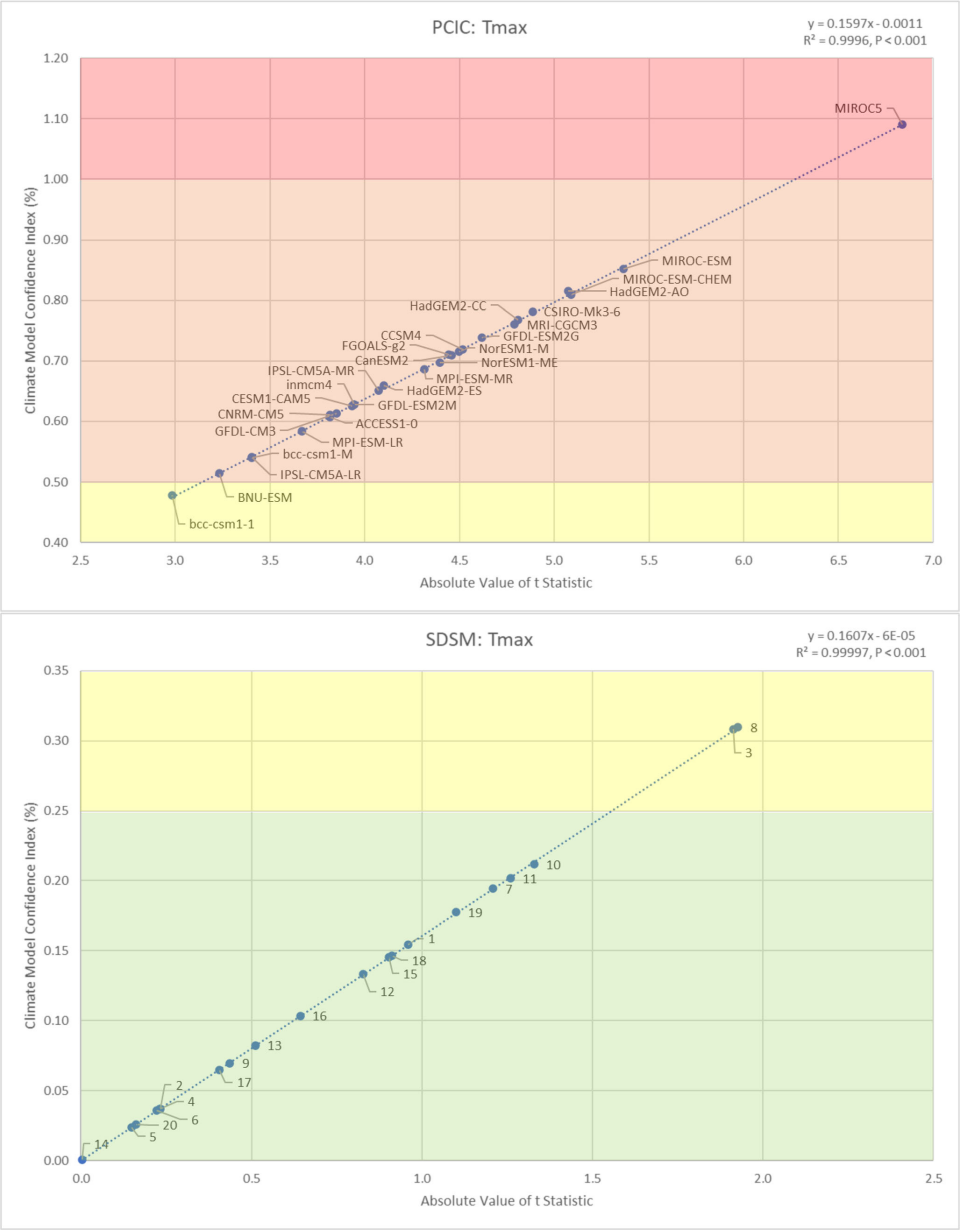
Comparing the ability of PCIC models and SDSM ensembles to reproduce baseline conditions (1969 to 2005) for maximum daily temperatures (*T*_max_) in Kelowna (BC), based on differences between means (│*t*│) and the CMCI

When evaluating the ability of PCIC models and SDSM ensembles to reproduce baseline conditions (1969 to 2005) for maximum daily temperature (*T*_max_) in Kelowna, BC (Fig. 1), it was apparent that the SDSM ensembles considerably outperformed the PCIC models. To such a degree that the worst SDSM ensembles still outperformed the best PCIC models. Most of the PCIC models (25 of 27) were characterised with “poor” performance (CMCI: 0.51 to 1.00), with only one model being considered “satisfactory” (CMCI: 0.26 to 0.50) and one model being considered “unacceptable” (CMCI > 1.00). In comparison, most of the SDSM ensembles (18 of 20) were characterised with “good” performance (CMCI: 0.00 to 0.25), with the remaining two ensembles being considered “satisfactory” (CMCI: 0.26 to 0.50). Nonetheless, it is interesting to note that of all PCIC climate models, the Beijing Climate Center’s Climate System Model (BCC-CSM version 1.1) was most capable (CMCI: 0.48) of reproducing baseline conditions for *T*_max_ in Kelowna. However, the modelled mean was 0.4 °C cooler than the observed mean, a difference that was statistically significant (*t* = −2.984, *P* < 0.001). In comparison, the best performing SDSM ensemble recorded a CMCI value of 0.0005, generating a modelled mean only 0.0004 °C warmer than the observed mean, a difference that was not statistically significant (*t* = 0.003, *P* = 0.499). It is also worthy to note that the twenty SDSM ensembles had a much smaller range of performance (CMCI: 0.00 to 0.31), compared with the larger range of CMCI values associated with the twenty-seven PCIC models (0.48 to 1.09). Finally, the SDSM ensembles also did a better job reproducing the observed variability of *T*_max_ in Kelowna, compared with the PCIC models (see Appendix Tables 1 and 2 for full results of all statistical tests for *T*_max_). In this regard, 18 of the 27 PCIC models (67%) reported statistically significant differences between the modelled and observed variances, compared with only 2 of the 20 SDSM ensembles (10%); these results are based on ANOVA *F*-tests for differences between variances, considering the 95% confidence level (*P* > 0.05).

### Minimum temperature

**Fig. 2:**
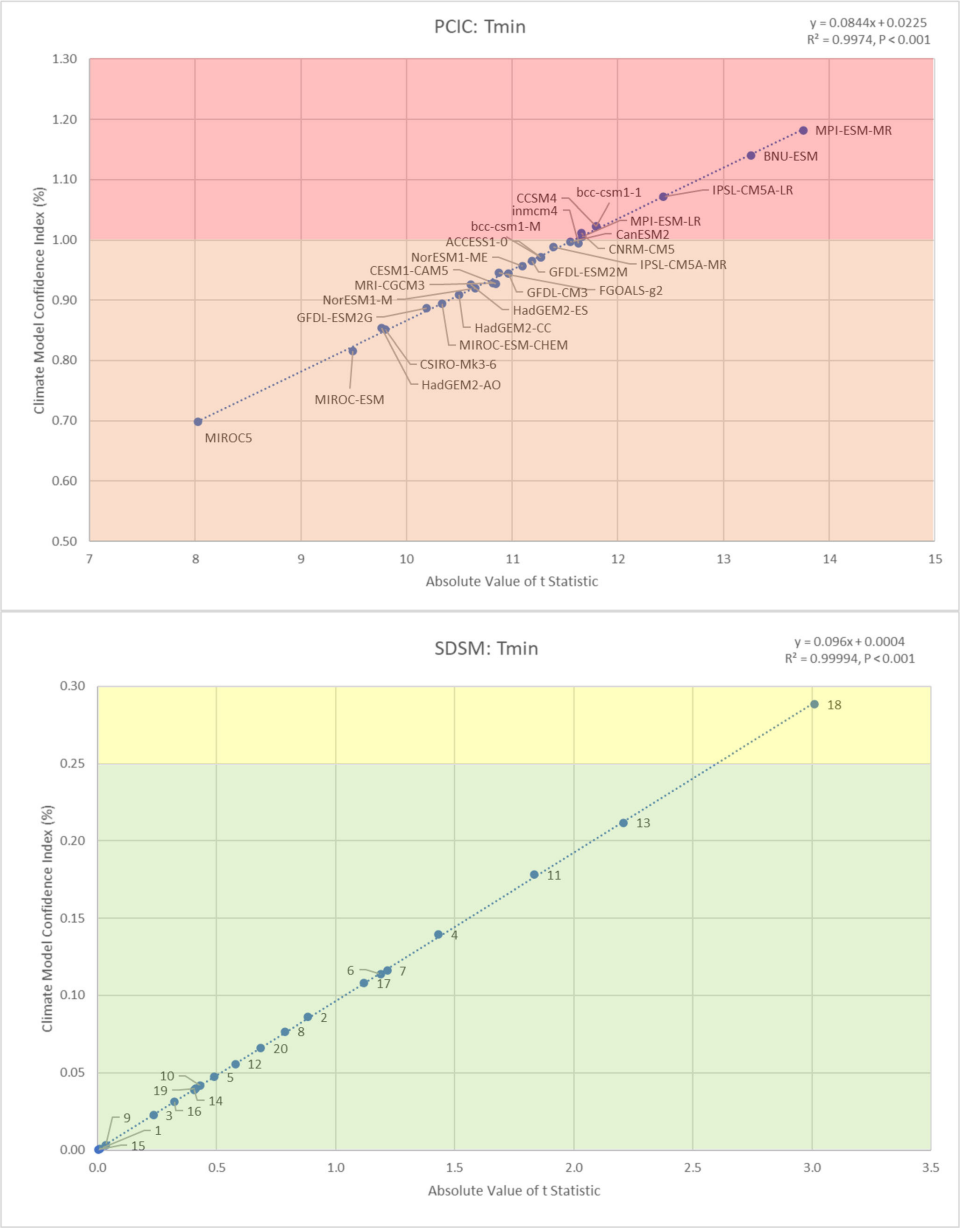
Comparing the ability of PCIC models and SDSM ensembles to reproduce baseline conditions (1969 to 2005) for minimum daily temperatures (*T*_min_) in Kelowna (BC), based on differences between means (│*t*│) and the CMCI

Looking at the ability of PCIC models and SDSM ensembles to reproduce baseline conditions for minimum daily temperatures (*T*_min_) in Kelowna, BC (Fig. 2), it was evident that the SDSM ensembles considerably outperformed the PCIC models, once again. As was the case for *T*_max_, so it is for *T*_min_ as well: even the worst SDSM ensembles outperformed the best PCIC models. Most of the PCIC models (18 of 27) were characterised with “poor” performance (CMCI: 0.51 to 1.00), while the remaining nine models were considered “unacceptable” (CMCI > 1.00), whereas most of the SDSM ensembles (19 of 20) were characterised with “good” performance (CMCI: 0.00 to 0.25), with the only remaining ensemble being considered “satisfactory” (CMCI: 0.26 to 0.50). Overall, both the PCIC models and the SDSM ensembles performed better for *T*_max_ than they did for *T*_min_, for this location. For example, at least one PCIC model was considered “satisfactory” for *T*_max_, while for *T*_min_, even the best PCIC model was still considered “poor”. Nonetheless, of all the PCIC climate models, the University of Tokyo’s Center for Climate System Research’s Model for Interdisciplinary Research On Climate (MIROC5), came closest to reproducing baseline conditions for *T*_min_ in Kelowna (CMCI: 0.70). However, the modelled mean was 0.7 °C warmer than the observed mean, a difference that was statistically significant (*t* = 8.023, *P* < 0.001). In comparison, the best performing SDSM ensemble for *T*_min_ recorded a CMCI value of 0.0002, generating a modelled mean only 0.0002 °C cooler than the observed mean, a difference that was not statistically significant (*t* = −0.002, *P* = 0.499). While the PCIC models performed considerably better for *T*_max_ than they did for *T*_min_, the SDSM ensembles performed similarly for both temperature variables, with performance being only slightly better for *T*_min_ than for *T*_max_. Furthermore, the twenty SDSM ensembles had a much smaller range of performance (CMCI: 0.00 to 0.28), compared with much higher and larger range of CMCI values associated with the twenty-seven PCIC models (0.70 to 1.18). Finally, the PCIC models did a better job of reproducing the observed variability of minimum daily temperatures in Kelowna, performing much better than they did for *T*_max_ and also slightly outperforming the SDSM ensembles for *T*_min_ (see Appendix Tables 3 and 4 for results of all statistical tests for *T*_min_). More specifically, only 11 of the 27 PCIC models (41%) reported statistically significant differences between the modelled and observed variance for *T*_min_, compared with 9 of the 20 SDSM ensembles (45%) for *T*_min_.

### Total precipitation

**Fig. 3:**
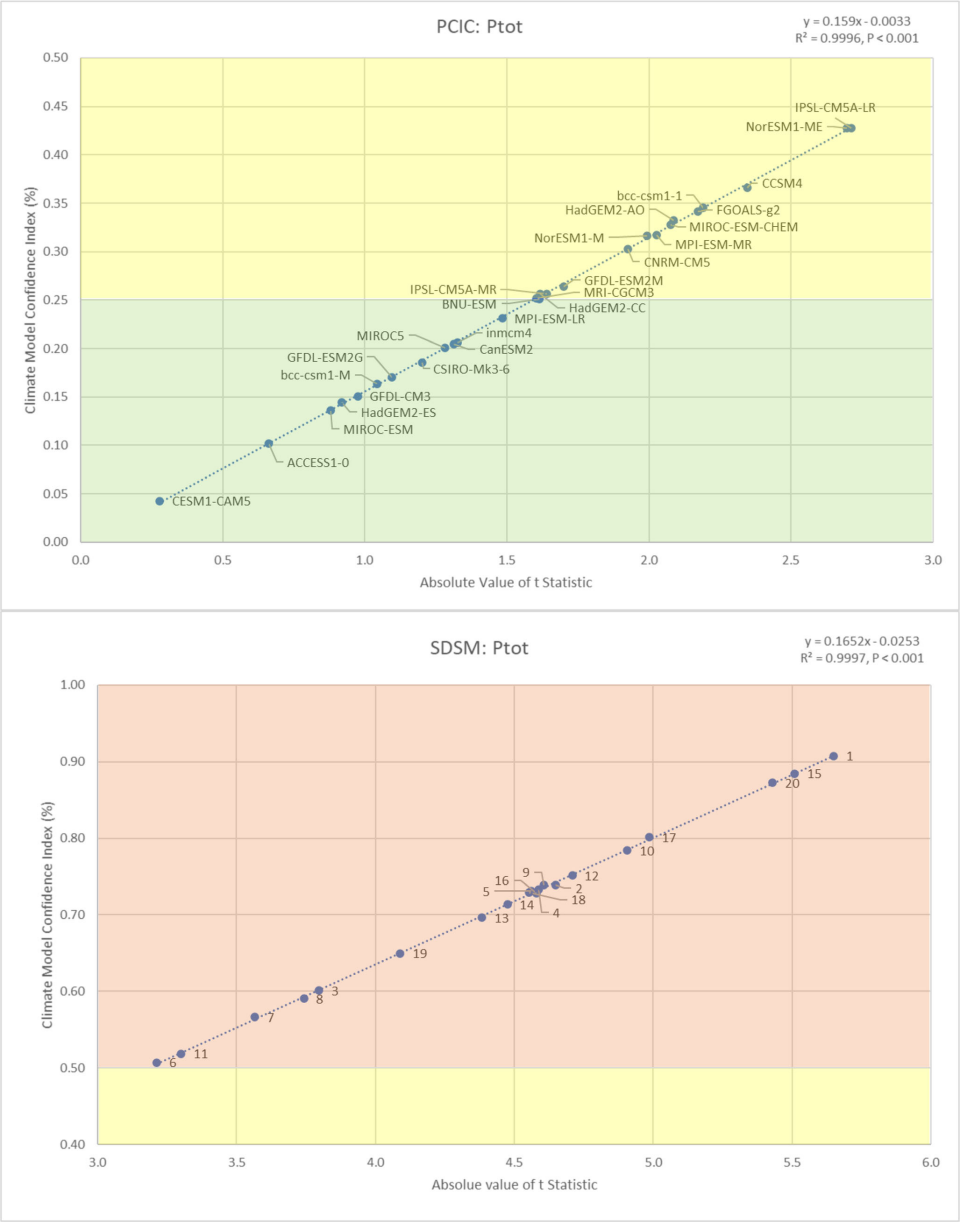
Comparing the ability of PCIC models and SDSM ensembles to reproduce baseline conditions (1969 to 2005) for total daily precipitation (*P*_tot_) in Kelowna (BC), based on differences between means (│*t*│) and the CMCI

Considering the ability of PCIC models and SDSM ensembles to reproduce baseline conditions for total daily precipitation (*P*_tot_) in Kelowna, BC (Fig. 3), this time, the PCIC models considerably outperformed the SDSM ensembles. For *P*_tot_, the worst PCIC models still outperformed the best SDSM ensembles. In this case, a slight majority of the PCIC models (14 of 27) were characterised with “good” performance (CMCI: 0.00 to 0.25), while the remaining 13 models were considered “satisfactory” (CMCI: 0.26 to 0.50), whereas all twenty SDSM ensembles were characterised by “poor” performance (CMCI: 0.51 to 1.00). Of all the PCIC climate models, the US National Center for Atmospheric Research’s Community Earth System Model version 1 including the Community Atmospheric Model version 5 (CESM1-CAM5) was most capable of reproducing baseline conditions for *P*_tot_ in Kelowna (CMCI: 0.04). Although the modelled mean was 0.01 mm wetter than the observed mean, this difference was not statistically significant (*t* = 0.277, *P* = 0.391). Whereas the best performing SDSM ensemble recorded a CMCI value of 0.51, generating a modelled mean 0.1 mm wetter than the observed mean, a difference that was statistically significant (*t* = 3.212, *P* = 0.001). The 27 PCIC models and the 20 SDSM ensembles were both associated with a similar range of performance based on CMCI values (0.04 to 0.43 and 0.51 to 0.91, respectively), but with the SDSM ensembles having much higher values. Finally, it is worthy to note that neither the PCIC modes nor SDSM ensembles were able to reproduce the observed variability of *P*_tot_, with all the models and ensembles reporting statistically significant differences between observed and modelled variances (see Appendix Tables 5 and 6 for full results of all the statistical tests for *P*_tot_).

### CMCI validation

For all climate variables considered (*T*_max_, *T*_min_ and *P*_tot_), regardless of the statistical downscaling technique employed (PCIC or SDSM), it was found that the CMCI values shared a nearly perfect correlation (*R*^2^ > 0.99, *P* < 0.001) with │*t*│ (Figs. 1, 2 and 3). Furthermore, there was a strong agreement in the order of magnitude between CMCI values and │*t*│, which was accurate up to the second decimal place (Appendices 1, 2 and 3). However, since the correlation between the CMCI and │*t*│ was not entirely perfect (*R*^2^ = 1), there was some confusion in the order of magnitude, with the discrepancy typically occurring at the third decimal place. Therefore, the *t* test remains the more accurate measure for ranking climate model performance based on the ability to reproduce average climate conditions, when compared with the CMCI. However, the calculation of *t* statistics in this case required daily data, while the CMCI was calculated using aggregated annual data, clearly demonstrating the utility of such a metric, while still remaining highly accurate (only 14 out of 141 model runs were ranked one position higher by CMCI than by │*t*│). Thus, the CMCI was shown to be an effective and efficient tool for evaluating and ranking the ability of climate models for reproducing local historical climate conditions. When discrepancies between │*t*│ and the CMCI value were observed, the corresponding *F* statistic gave us the clarifying information we initially expected, but the direction of influence we had hoped for was inverted. The model which received a one position higher rank according to the CMCI was always associated with a higher *F* statistic than the model that received the lower rank, indicting that │*t*│ more accurately identifies and ranks models regarding both differences between means and differences between variances. The reason for this discrepancy is because the *t* test considers both observed and modelled variance within its calculation while the CMCI only considers observed variance. Furthermore, the better the models/ensembles performed at reproducing baseline climate conditions within a variable grouping (e.g. SDSM temperature ensembles), the more accurate the CMCI was in comparison with │*t*│. To such a degree that most of the discrepancies between CMCI values and │*t*│ were observed among the PCIC temperature models, which were also associated with the most statistically significant differences between modelled and observed means, whereas the only discrepancies reported among the SDSM ensemble rankings were for precipitation, the variable that SDSM performed the worst for.

## Discussion and conclusions

This study further develops and validates the CMCI previously published within several CCIAs (Hewer and Gough 2016a, b, 2019a, b, 2020; Hewer and Brunette 2020) as an effective and efficient tool for measuring and ranking the ability of climate models to reproduce historical climate conditions. This was accomplished by comparing statistically downscaled baseline climate scenarios from PCIC models and SDSM ensembles in Kelowna, BC (Canada) for the period from 1969 to 2005. Student’s *t* tests (*N* > 13,000) were used to determine if there were statistically significant differences between modelled and observed means on the daily time scale; then, the CMCI was calculated using aggregated annual values to validate this metric for evaluating and ranking climate model performance. It was shown that the CMCI values were in close agreement with the rank order of │*t*│ (99% confidence level). Furthermore, of the 141 rankings determined, 127 model runs (90.1%) were ordered in the same way as the │*t*│ rankings and the 14 model runs that were in discrepancy were only slightly different (realised at the third decimal place and resulting in the model being ranked one position higher than the ranking according to │*t*│). Finally, it was determined that this discrepancy was explained by differences between the observed and modelled variances, as illustrated by the resulting *F* statistic. The *t* test considers both modelled and observed variance within its calculation, whereas the CMCI only considers (and requires) observed variance for its calculation. Although this is a slight shortcoming relating to precision, it still demonstrates increased utility since the CMCI can be calculated without requiring modelled variance, which is often not readily available to researchers trying to determine which climate models to use in an impact assessment, especially when considering the wide array of GCMs available from CMIP5 (Taylor et al. 2012). The utility of this metric is further seen in that the CMCI is easy to calculate, operates with readily available climate inputs (annual data) and small sample sizes (*N* ≥ 30), while the more sophisticated Student’s *t* tests are more effective with larger sample sizes, which often demands access to monthly or daily data, and typically requires some form of statistical software for calculation.

Previously, the CMCI has been employed to evaluate and rank GCMs in an effort to create multi-model ensembles made up of the best performing GCMs, referred to as the selective ensemble approach (Hewer and Gough 2016a, b, 2019a, b), an alternative to the more common full ensemble approach (Fenech et al. 2007; IPCC 2010; Hewer et al. 2016). In this study, the CMCI has also been shown to be an effective tool for evaluating, ranking, and selecting individual model runs from statistically downscaled climate scenarios. This is another important utility that the CMCI offers, especially for localised climate change impact assessments that require daily data for the assessment (Hewer and Gough 2020; Hewer and Brunette 2020); in that, these studies should select one scenario to base the assessment upon, since averaging multiple daily scenarios eliminates day-to-day variability associated with local climatology (Gough 2008). For example, if a study elected to average the daily output from all the available PCIC models or from all the available SDSM ensembles, then the occurrence of extreme temperature events would be considerably diminished and essentially every day in the scenario would show average conditions. This concern is exacerbated with precipitation scenarios as each day would then record some small amount of rain rather than having many days with no rain and other days with varying amounts of rain (from trace amounts up to heavy rain events). Developing and validating a metric to aid researchers in ranking and selecting which statistically downscaled climate change scenario to use within a CCIA has even greater utility within the field of applied climatology when considering critical thresholds associated with daily weather events (Vincent et al. 2018; Hewer 2020). Some further examples of potential applications include thresholds and indicators associated with climatic suitability for agriculture (Caprio and Quamme 1999, 2002, 2006; Rayne and Forest 2016); the survival of pests and insects (Campbell et al. 2014); thermal stress on human health due to extreme temperatures (Casati et al. 2013); or the effect of freeze thaw events on road maintenance (Ho and Gough 2006).

The main limitation associated with the CMCI is that it was shown to only be accurate up to the second decimal place and if the CMCI was rounded to the second decimal place to smooth out any discrepancies occurring at the third decimal place, then on those occasions, the models would be ranked with a tie, which was never the case for the more precise *t* statistic. Also, the worse that the models being evaluated performed at reproducing local historical climate conditions, the more prone to error the CMCI became. However, it is worthy to note that the CMCI was still always able to effectively determine the particular model/ensemble that performed best (no ties or discrepancies reported among the top models for each climate variable within this study), thus supporting its effective use in the selection of which climate scenario to use within an impact assessment. More generally, further development and validation of the CMCI is also limited by the scope of this study that only considered one location (Kelowna, BC), which is also associated with a unique microclimate within the Okanagan Valley (Rayne and Forest 2016). Therefore, future research is required to expand this analysis across other locations (Sobie and Murdock 2017), to confirm the findings of this study with more data and with more site-specific applications.

Although it was not the primary purpose of the paper, some conclusions can also be drawn about the performance of the two different statistical downscaling techniques employed in this study. In the case of Kelowna, BC (Canada), the results of this study suggest that SDSM was more effective at reproducing local temperature conditions from 1969 to 2005, while PCIC was more effective at reproducing local precipitation conditions. This could be explained by the parametric nature of the SDSM modelling approach (Wilby et al. 2002) compared with the nonparametric quantile mapping approach of PCIC (Gudmundsson et al. 2012; Hunter and Meentemeyer 2005). Furthermore, it could be argued that SDSM is a more appropriate method for localised assessments since it is calibrated with weather station data (Wilby and Dawson 2013; Eum et al. 2014), while PCIC may be more appropriate for regional assessments, since it is calibrated with 10-km gridded climate data (McKenney et al. 2011; Hopkinson et al. 2011). It is also important to note that SDSM is a skill-based program whose output is dependent upon the ability of the user, while PCIC is a point and click platform that should generate the same quality of output regardless of the user’s skill. Therefore, other researchers may generate better of worse SDSM models in comparison with those analysed in this study, which could skew the results in comparison with the performance of the PCIC models, which should remain constant (assuming the same grid box is properly selected for download). Finally, to draw more generalizable conclusions about the performance of SDSM compared with PCIC, additional study sites across diverse climates would be required and is therefore an important area of future research.

## Appendix

**Table 1:** PCIC models for *T*_max_

*T*_max_ PCIC model	Var. Mod.	Var. Obs.	*F*	*P*(*F*)	Mean Mod.	Mean Obs.	*t*	*P*(*t*)	CMCI
bcc-csm1-1	117.3	118.9	0.987	0.217	13.72	14.12	*− 2.984*	*0.001*	0.478
BNU-ESM	114.9	118.9	*0.967*	*0.024*	13.69	14.12	*− 3.231*	*0.001*	0.515
IPSL-CM5A-LR	114.2	118.9	*0.961*	*0.010*	13.67	14.12	*− 3.403*	*0.000*	0.542
bcc-csm1-M	112.5	118.9	*0.946*	*0.001*	13.67	14.12	*− 3.404*	*0.000*	0.540
MPI-ESM-LR	114.9	118.8	*0.967*	*0.025*	13.63	14.11	*− 3.669*	*0.000*	0.585
CNRM-CM5	116.7	118.8	0.982	0.150	13.61	14.11	*− 3.817*	*0.000*	0.610
ACCESS1-0	114.5	118.8	*0.964*	*0.016*	13.61	14.11	*− 3.818*	*0.000*	0.608
GFDL-CM3	114.8	118.9	*0.966*	*0.021*	13.61	14.12	*− 3.851*	*0.000*	0.614
CESM1-CAM5	113.8	118.9	*0.958*	*0.006*	13.60	14.12	*− 3.933*	*0.000*	0.625
GFDL-ESM2M	114.6	118.9	*0.964*	*0.017*	13.60	14.12	*− 3.940*	*0.000*	0.628
inmcm4	114.4	118.9	*0.962*	*0.013*	13.60	14.12	*− 3.943*	*0.000*	0.628
IPSL-CM5A-MR	115.7	118.9	0.973	0.056	13.58	14.12	*− 4.075*	*0.000*	0.650
HadGEM2-ES	116.6	117.8	0.990	0.275	13.56	14.10	*− 4.100*	*0.000*	0.659
MPI-ESM-MR	114.0	118.8	*0.959*	*0.008*	13.55	14.11	*− 4.316*	*0.000*	0.686
NorESM1-ME	113.3	118.9	*0.953*	*0.003*	13.54	14.12	*− 4.394*	*0.000*	0.698
FGOALS-g2	116.9	118.9	0.983	0.164	13.53	14.12	*− 4.441*	*0.000*	0.711
CanESM2	114.0	118.9	*0.959*	*0.008*	13.53	14.12	*− 4.458*	*0.000*	0.709
NorESM1-M	114.5	118.9	*0.963*	*0.015*	13.53	14.12	*− 4.496*	*0.000*	0.716
CCSM4	114.5	118.9	*0.964*	*0.015*	13.53	14.12	*− 4.515*	*0.000*	0.719
GFDL-ESM2G	116.8	118.9	0.983	0.156	13.51	14.12	*− 4.618*	*0.000*	0.739
MRI-CGCM3	113.0	118.8	*0.951*	*0.002*	13.49	14.11	*− 4.791*	*0.000*	0.760
HadGEM2-CC	113.5	117.8	*0.964*	*0.016*	13.47	14.10	*− 4.807*	*0.000*	0.768
CSIRO-Mk3-6	116.6	118.9	0.981	0.135	13.47	14.12	*− 4.885*	*0.000*	0.781
HadGEM2-AO	116.0	118.1	0.982	0.152	13.40	14.07	*− 5.075*	*0.000*	0.815
MIROC-ESM-CH	114.3	118.8	*0.962*	*0.012*	13.45	14.11	*− 5.088*	*0.000*	0.810
MIROC-ESM	113.5	118.8	*0.955*	*0.004*	13.41	14.11	*− 5.366*	*0.000*	0.852
MIROC5	115.6	118.9	0.972	0.051	13.22	14.12	*− 6.836*	*0.000*	1.091

Values in italics indicate that differences were statistically significant at the 95% confidence level (*P *< 0.05)

**Table 2:** SDSM ensembles for *T*_max_

*T*_max_ SDSM ensemble	Var. Mod.	Var. Obs.	*F*	*P*(*F*)	Mean Mod.	Mean Obs.	*t*	*P*(*t*)	CMCI
14	118.9	118.8	1.001	0.482	14.11	14.11	0.003	0.499	0.000
5	118.0	118.8	0.993	0.335	14.09	14.11	− 0.148	0.441	0.024
20	121.8	118.8	1.025	0.076	14.09	14.11	− 0.161	0.436	0.026
6	120.9	118.8	1.018	0.156	14.08	14.11	− 0.222	0.412	0.036
4	119.1	118.8	1.002	0.456	14.14	14.11	0.231	0.409	0.037
2	112.1	118.8	*0.943*	*0.000*	14.14	14.11	0.233	0.408	0.037
17	118.7	118.8	0.999	0.470	14.06	14.11	− 0.405	0.343	0.065
9	116.3	118.8	0.978	0.102	14.17	14.11	0.437	0.331	0.070
13	120.2	118.8	1.012	0.248	14.05	14.11	− 0.511	0.305	0.082
16	119.7	118.8	1.007	0.345	14.03	14.11	− 0.643	0.260	0.103
12	119.2	118.8	1.003	0.436	14.22	14.11	0.828	0.204	0.133
15	120.6	118.8	1.015	0.193	13.99	14.11	− 0.903	0.183	0.146
18	117.9	118.8	0.992	0.321	14.23	14.11	0.912	0.181	0.146
1	119.7	118.8	1.007	0.332	14.24	14.11	0.958	0.169	0.154
19	121.3	118.8	1.021	0.116	14.26	14.11	1.099	0.136	0.177
7	119.8	118.8	1.008	0.324	14.27	14.11	1.208	0.114	0.194
11	117.0	118.8	0.984	0.178	14.28	14.11	1.260	0.104	0.202
10	114.8	118.8	*0.966*	*0.022*	14.29	14.11	1.329	0.092	0.212
3	120.3	118.8	1.012	0.237	14.37	14.11	*1.912*	*0.028*	0.308
8	119.1	118.8	1.002	0.457	14.37	14.11	*1.927*	*0.027*	0.310

Values in italics indicate that differences were statistically significant at the 95% confidence level (*P* < 0.05)

**Table 3:** PCIC models for *T*_min_

*T*_min_ PCIC model	Var. Mod.	Var. Obs.	*F*	*P*(*F*)	Mean Mod.	Mean Obs.	*t*	*P*(*t*)	CMCI
MIROC5	58.1	57.9	1.004	0.407	2.21	1.47	*8.023*	*0.000*	0.698
MIROC-ESM	55.6	57.9	*0.961*	*0.011*	2.33	1.46	*9.490*	*0.000*	0.816
HadGEM2-AO	58.3	57.5	1.013	0.221	2.35	1.44	*9.761*	*0.000*	0.854
CSIRO-Mk3-6	57.8	57.9	0.999	0.479	2.37	1.47	*9.800*	*0.000*	0.851
GFDL-ESM2G	58.4	57.9	1.009	0.293	2.41	1.47	*10.188*	*0.000*	0.887
MIROC-ESM-CH	56.7	57.9	0.980	0.126	2.42	1.46	*10.339*	*0.000*	0.894
HadGEM2-CC	55.9	57.3	0.976	0.078	2.43	1.46	*10.495*	*0.000*	0.909
HadGEM2-ES	57.6	57.3	1.004	0.406	2.45	1.46	*10.608*	*0.000*	0.926
NorESM1-M	56.3	57.9	0.973	0.058	2.45	1.47	*10.654*	*0.000*	0.920
MRI-CGCM3	55.1	57.9	*0.953*	*0.002*	2.45	1.46	*10.818*	*0.000*	0.928
CESM1-CAM5	54.1	57.9	*0.935*	*0.000*	2.45	1.47	*10.848*	*0.000*	0.927
FGOALS-g2	58.2	57.9	1.005	0.380	2.47	1.47	*10.871*	*0.000*	0.946
GFDL-CM3	56.0	57.9	*0.968*	*0.029*	2.47	1.47	*10.963*	*0.000*	0.945
NorESM1-ME	56.0	57.9	*0.968*	*0.028*	2.49	1.47	*11.095*	*0.000*	0.956
GFDL-ESM2M	56.3	57.9	0.973	0.058	2.50	1.47	*11.187*	*0.000*	0.966
bcc-csm1-M	56.2	57.9	*0.971*	*0.001*	2.50	1.46	*11.269*	*0.000*	0.972
ACCESS1-0	56.2	57.9	*0.971*	*0.046*	2.50	1.46	*11.269*	*0.000*	0.972
IPSL-CM5A-MR	57.6	57.9	0.996	0.409	2.52	1.47	*11.389*	*0.000*	0.989
CanESM2	56.5	57.9	0.976	0.075	2.53	1.47	*11.548*	*0.000*	0.997
inmcm4	54.2	57.9	*0.937*	*0.000*	2.53	1.47	*11.625*	*0.000*	0.994
CNRM-CM5	57.8	57.9	0.999	0.467	2.54	1.46	*11.656*	*0.000*	1.012
MPI-ESM-LR	56.3	57.9	0.973	0.058	2.53	1.46	*11.659*	*0.000*	1.006
CCSM4	57.4	57.9	0.992	0.121	2.56	1.47	*11.795*	*0.000*	1.023
bcc-csm1-1	57.4	57.9	0.992	0.317	2.56	1.47	*11.795*	*0.000*	1.023
IPSL-CM5A-LR	56.0	57.9	*0.968*	*0.030*	2.61	1.47	*12.431*	*0.000*	1.072
BNU-ESM	55.4	57.9	*0.957*	*0.005*	2.68	1.47	*13.258*	*0.000*	1.140
MPI-ESM-MR	55.5	57.9	*0.958*	*0.007*	2.72	1.46	*13.754*	*0.000*	1.182

Values in italics indicate that differences were statistically significant at the 95% confidence level (*P* < 0.05)

**Table 4:** SDSM ensembles for *T*_min_

*T*_min_ SDSM ensemble	Var. Mod.	Var. Obs.	*F*	*P*(*F*)	Mean Mod.	Mean Obs.	*t*	*P*(*t*)	CMCI
15	55.9	57.9	*0.967*	*0.025*	1.46	1.46	− 0.002	0.499	0.000
1	58.7	57.9	1.015	0.192	1.46	1.46	0.009	0.497	0.001
9	58.9	57.9	1.018	0.148	1.46	1.46	− 0.034	0.486	0.003
3	56.2	57.9	*0.972*	*0.049*	1.48	1.46	0.234	0.407	0.023
16	58.2	57.9	1.005	0.377	1.43	1.46	− 0.323	0.373	0.031
14	56.0	57.9	*0.967*	*0.027*	1.50	1.46	0.406	0.342	0.039
19	57.5	57.9	0.994	0.361	1.50	1.46	0.413	0.340	0.040
10	59.8	57.9	*1.034*	*0.025*	1.42	1.46	− 0.429	0.334	0.042
5	57.7	57.9	0.997	0.436	1.42	1.46	− 0.491	0.312	0.047
12	57.3	57.9	0.990	0.273	1.41	1.46	− 0.578	0.282	0.056
20	56.3	57.9	0.972	0.052	1.53	1.46	0.685	0.247	0.066
8	59.6	57.9	*1.030*	*0.041*	1.39	1.46	− 0.787	0.216	0.077
2	58.7	57.9	1.015	0.197	1.38	1.46	− 0.884	0.188	0.086
17	57.4	57.9	0.992	0.323	1.57	1.46	1.119	0.132	0.108
6	55.2	57.9	*0.954*	*0.003*	1.57	1.46	1.189	0.117	0.114
7	54.8	57.9	*0.947*	*0.001*	1.57	1.46	1.216	0.112	0.116
4	59.4	57.9	1.027	0.059	1.33	1.46	− 1.431	0.076	0.139
11	58.6	57.9	1.012	0.242	1.29	1.46	*− 1.835*	*0.033*	0.178
13	55.5	57.9	*0.959*	*0.008*	1.67	1.46	*2.209*	*0.014*	0.212
18	55.3	57.9	*0.955*	*0.004*	1.74	1.46	*3.011*	*0.001*	0.288

Values in italics indicate that differences were statistically significant at the 95% confidence level (*P* < 0.05)

**Table 5:** PCIC models for *P*_tot_

*P*_tot_ PCIC model	Var. Mod.	Var. Obs.	*F*	*P*(*F*)	Mean Mod.	Mean Obs.	*t*	*P*(*t*)	CMCI
CESM1-CAM5	5.01	6.38	*0.785*	*0.000*	1.02	1.01	0.277	0.391	0.043
ACCESS1-0	4.98	6.37	*0.782*	*0.000*	1.03	1.01	0.662	0.254	0.102
MIROC-ESM	5.11	6.37	*0.801*	*0.000*	1.04	1.01	0.880	0.189	0.137
HadGEM2-ES	5.14	6.43	*0.800*	*0.000*	1.04	1.01	0.919	0.179	0.144
GFDL-CM3	5.05	6.38	*0.792*	*0.000*	1.04	1.01	0.975	0.165	0.151
bcc-csm1-M	5.32	6.38	*0.835*	*0.000*	1.04	1.01	1.043	0.149	0.163
GFDL-ESM2G	5.19	6.38	*0.814*	*0.000*	1.04	1.01	1.095	0.137	0.170
CSIRO-Mk3-6	5.06	6.38	*0.794*	*0.000*	1.05	1.01	1.201	0.115	0.186
MIROC5	5.30	6.38	*0.832*	*0.000*	1.05	1.01	1.281	0.100	0.200
CanESM2	5.20	6.38	*0.816*	*0.000*	1.05	1.01	1.313	0.095	0.205
inmcm4	5.18	6.38	*0.812*	*0.000*	1.05	1.01	1.326	0.092	0.206
MPI-ESM-LR	5.22	6.37	*0.818*	*0.000*	1.05	1.01	1.484	0.069	0.231
MRI-CGCM3	5.37	6.37	*0.843*	*0.000*	1.06	1.01	1.605	0.054	0.252
BNU-ESM	5.19	6.38	*0.814*	*0.000*	1.06	1.01	1.614	0.053	0.251
HadGEM2-CC	5.41	6.43	*0.841*	*0.000*	1.06	1.01	1.616	0.053	0.257
IPSL-CM5A-MR	5.27	6.38	*0.826*	*0.000*	1.06	1.01	1.640	0.051	0.256
GFDL-ESM2M	5.14	6.38	*0.807*	*0.000*	1.06	1.01	*1.700*	*0.045*	0.264
CNRM-CM5	5.42	6.37	*0.851*	*0.000*	1.07	1.01	*1.927*	*0.027*	0.303
NorESM1-M	5.64	6.38	*0.885*	*0.000*	1.07	1.01	*1.992*	*0.023*	0.316
MPI-ESM-MR	5.32	6.37	*0.834*	*0.000*	1.07	1.01	*2.027*	*0.021*	0.317
MIROC-ESM-CH	5.50	6.37	*0.863*	*0.000*	1.07	1.01	*2.077*	*0.019*	0.328
HadGEM2-AO	5.54	6.42	*0.862*	*0.000*	1.08	1.01	*2.086*	*0.018*	0.333
FGOALS-g2	5.45	6.38	*0.854*	*0.000*	1.08	1.01	*2.171*	*0.015*	0.342
bcc-csm1-1	5.45	6.38	*0.855*	*0.000*	1.08	1.01	*2.192*	*0.014*	0.345
CCSM4	5.21	6.38	*0.817*	*0.000*	1.08	1.01	*2.347*	*0.009*	0.366
NorESM1-ME	5.63	6.38	*0.883*	*0.000*	1.09	1.01	*2.696*	*0.004*	0.428
IPSL-CM5A-LR	5.51	6.38	*0.863*	*0.000*	1.09	1.01	*2.711*	*0.003*	0.696

Values in italics indicate that differences were statistically significant at the 95% confidence level (*P* < 0.05)

**Table 6:** SDSM ensembles for *P*_tot_

*P*_tot_ SDSM ensemble	Var. Mod.	Var. Obs.	*F*	*P*(*F*)	Mean Mod.	Mean Obs.	*t*	*P*(*t*)	CMCI
6	5.52	6.37	*0.866*	*0.000*	1.11	1.01	*3.212*	*0.001*	0.507
11	5.42	6.37	*0.851*	*0.000*	1.11	1.01	*3.300*	*0.000*	0.519
7	5.67	6.37	*0.889*	*0.000*	1.12	1.01	*3.567*	*0.000*	0.566
8	5.52	6.37	*0.866*	*0.000*	1.12	1.01	*3.742*	*0.000*	0.591
3	5.60	6.37	*0.879*	*0.000*	1.12	1.01	*3.796*	*0.000*	0.601
19	5.68	6.37	*0.891*	*0.000*	1.13	1.01	*4.089*	*0.000*	0.650
13	5.66	6.37	*0.889*	*0.000*	1.14	1.01	*4.384*	*0.000*	0.696
14	5.77	6.37	*0.905*	*0.000*	1.14	1.01	*4.475*	*0.000*	0.714
18	5.86	6.37	*0.920*	*0.000*	1.15	1.01	*4.553*	*0.000*	0.729
5	5.88	6.37	*0.923*	*0.000*	1.15	1.01	*4.562*	*0.000*	0.731
16	5.70	6.37	*0.894*	*0.000*	1.15	1.01	*4.579*	*0.000*	0.728
4	5.80	6.37	*0.911*	*0.000*	1.15	1.01	*4.587*	*0.000*	0.733
9	5.90	6.37	*0.926*	*0.000*	1.15	1.01	*4.605*	*0.000*	0.738
2	5.68	6.37	*0.891*	*0.000*	1.15	1.01	*4.648*	*0.000*	0.739
12	5.77	6.37	*0.906*	*0.000*	1.15	1.01	*4.709*	*0.000*	0.751
10	5.80	6.37	*0.911*	*0.000*	1.16	1.01	*4.907*	*0.000*	0.784
17	5.95	6.37	*0.934*	*0.000*	1.16	1.01	*4.986*	*0.000*	0.801
20	5.95	6.37	*0.933*	*0.000*	1.17	1.01	*5.430*	*0.000*	0.872
15	5.94	6.37	*0.931*	*0.000*	1.18	1.01	*5.509*	*0.000*	0.885
1	5.95	6.37	*0.934*	*0.000*	1.18	1.01	*5.650*	*0.000*	0.908

Values in italics indicate that differences were statistically significant at the 95% confidence level (*P* < 0.05)
